# Combined Pharmacological Modulation of Translational and Transcriptional Activity Signaling Pathways as a Promising Therapeutic Approach in Children with Myocardial Changes

**DOI:** 10.3390/biom14040477

**Published:** 2024-04-13

**Authors:** Andrii Kamenshchyk, Igor Belenichev, Valentyn Oksenych, Oleksandr Kamyshnyi

**Affiliations:** 1 Department of Hospital Pediatrics, Zaporizhzhya State Medical and Pharmaceutical University, 69035 Zaporizhzhya, Ukraine; 2 Department of Pharmacology, Zaporizhzhya State Medical and Pharmaceutical University, 69035 Zaporizhzhya, Ukraine; i.belenichev1914@gmail.com; 3 Broegelmann Research Laboratory, Department of Clinical Science, University of Bergen, 5020 Bergen, Norway; 4 Department of Microbiology, Virology and Immunology, I. Horbachevsky Ternopil State Medical University, 46001 Ternopil, Ukraine; alexkamyshnyi@gmail.com

**Keywords:** myocardial hypertrophy, children, gene expression signaling pathways, pharmacological modulation, heart

## Abstract

Myocardial hypertrophy is the most common condition that accompanies heart development in children. Transcriptional gene expression regulating pathways play a critical role both in cardiac embryogenesis and in the pathogenesis of congenital hypertrophic cardiomyopathy, neonatal posthypoxic myocardial hypertrophy, and congenital heart diseases. This paper describes the state of cardiac gene expression and potential pharmacological modulators at different transcriptional levels. An experimental model of perinatal cardiac hypoxia showed the downregulated expression of genes responsible for cardiac muscle integrity and overexpressed genes associated with energy metabolism and apoptosis, which may provide a basis for a therapeutic approach. Current evidence suggests that RNA drugs, theaflavin, neuraminidase, proton pumps, and histone deacetylase inhibitors are promising pharmacological agents in progressive cardiac hypertrophy. The different points of application of the above drugs make combined use possible, potentiating the effects of inhibition in specific signaling pathways. The special role of N-acetyl cysteine in both the inhibition of several signaling pathways and the reduction of oxidative stress was emphasized.

## 1. Introduction

**Figure 1:**
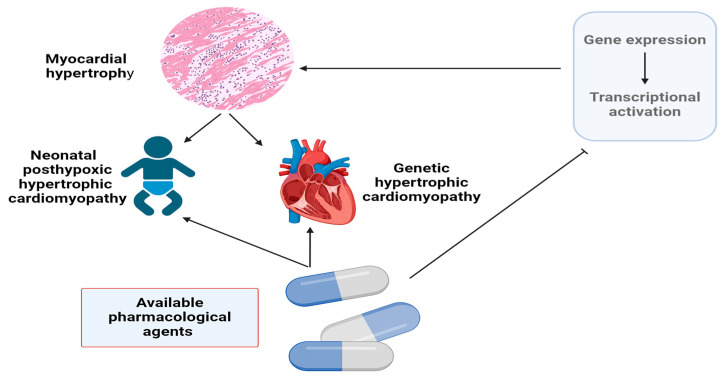
The study’s search algorithm is illustrated by a flow chart showing the relationship between gene expression with subsequent activation of transcriptional pathways and common pediatric hypertrophic heart diseases. The image was made using BioRender (accessed on 24 March 2024).

Transcription factors play a critical role in the progression of myocardial changes, including hypertrophy, fibrosis, genetic cardiomyopathy, and neonatal posthypoxic hypertrophic cardiomyopathy. Recently, many studies have demonstrated the potential to regulate gene expression signaling pathways [1,2,3]. Expression of transcription-activating genes plays a critical role in the myocardium, not only in cardiac hypertrophy but also in intrauterine cardiac differentiation and valve formation [4,5]. Consistent with this, our previous studies have shown that children with bicuspid aortic valve (BAV) overexpress a nuclear factor of the activated T-cells 1 (NFATC1) gene, the key transcriptional activator, which correlates with the left ventricular hypertrophy [6]. On the other hand, many substances that may be able to inhibit cardiac hypertrophy by this mechanism are currently in various stages of clinical trials. Some are components of gene therapy and are not yet applicable in pediatric practice. It should also be emphasized that the ability of already known and relatively safe pharmaceutical agents to modulate the activity of transcriptional and translational factors in the heart at different levels is not sufficiently exploited in practice. The type and duration of hypertrophic stimuli determine the transition from adaptive or physiological hypertrophy to pathological changes in the heart [7]. This is of particular importance in children with high adaptive potential and with a predominantly progressive and asymptomatic course of myocardial changes. Therefore, our study aimed to analyze the current situation regarding the possibility of existing drugs that could potentially be used in children to influence the translational and transcriptional pathways involved in cardiac hypertrophy. To achieve this, we analyzed the expression of genes and their signaling pathways regarding the most common primary hypertrophic heart conditions in children (Figure 1). We then identified and assessed the applicability of the pharmaceutical agents that can affect gene expression pathways at different levels.

## 2. Regulatory Factors of Gene Expression in Myocardial Hypertrophy and Approaches to Pharmacological Modulation

Experiments with human heart failure cardiomyocytes and transaortic banding have shown an increase in gene expression in adaptive myocardial hypertrophy and a decrease in gene expression in pathological hypertrophy. Overexpressed cardiac stress genes, such as NPPB (natriuretic peptide) and MYH7 (beta-myosin heavy chain), were identified. At the same time, a downregulation of almost all mitochondrial genes in maladaptive cardiomyocytes reflects reduced transcriptional activity. However, RASL11B, a small GTPase protein with apoptotic activity in cancer cells, was found to be overexpressed in cardiomyocytes and to play a special role in increasing the activity of glycolytic enzyme genes, such as PFKP (platelet isoform of phosphofructokinase) in failing cardiomyocytes. Thus, the authors have proposed inhibition of therapeutic activity [8]. Calcineurin, a Ca^2+^-dependent serine/threonine protein phosphatase, was found to have main functions in the development of myocardial hypertrophy. It causes internalization of NFATC into the nucleus of cardiomyocytes with consequent transcriptional activation [9,10]. The long noncoding RNA (lncRNA) gene H19, which is downregulated in hypertrophic hearts and activated by viral-based gene therapy, suppresses pro-hypertrophic NFATC signaling and reverses pathological myocardial hypertrophy by restoring lncRNAs [11,12]. Using samples from failing hearts and aortic banding mice, nuclear-localized protein 1 was shown to negatively regulate cardiac hypertrophy by suppressing NFATC3 transcription independently of calcineurin activity [13]. Theaflavin-3,3′-digallate (TF3), the main constituent of black tea, was found to reduce calcineurin (CaN) levels and increase p-NFATc3 protein expression. It suggests that TF3 inhibits the transmission of the hypertrophy signal, at least in part, through the CaN-NFAT pathway by reducing Ca^2+^ levels. In cardiomyocytes with pathological hypertrophy, TF3 binds to both calmodulin and CaN, leading to downregulation and, ultimately, inhibition of CaN-NFAT pathway activation [14].

The balance between acetylation and deacetylation of histones by the enzymes histone acetylase (HAT) and histone deacetylase (HDAC), correspondingly, leads to post-translational modification of histones, and HDAC1 downregulates the expression of genes that inhibit cardiac hypertrophy. On the contrary, HDAC2, by binding to NFAT or GATA4, activates excessive gene expression, which has led to the development of myocardial hypertrophy. Short-chain fatty acids are weak NFATC inhibitors with moderate selectivity for HDAC1; however, hydroxamic acid has the most zinc-chelating properties compared to other inhibitors, and it binds specifically to all types of HDACs. Simultaneously, benzamide and cyclic peptides are highly selective for HDAC 1–3 [15]. In a culture of human umbilical vein endothelial cells, valproic acid, as an HDAC inhibitor, triggers a 5–6-fold expression increase in the calcium-activated potassium channel (KCa2.3) and potentiates vasodilation [16]. In general, HDAC inhibitors act through Zn^2+^ binding and exert their protective effects in myocardial hypertrophy and arterial hypertension, myocardial infarction, and atrial fibrillation [17].

Overexpression of neuraminidase 1 (NEU1) is observed in cardiomyocytes, invading monocytes, and leads to inflammation, heart hypertrophy, and heart failure in ischemia-reperfusion heart injury in mice. The authors proposed specific inhibition of NEU1 as a promising therapeutic strategy [18]. The overexpression of neuraminidase 1 in hypertrophic cardiomyocytes and its interaction with the nuclear GATA4 gene promotes the development of cardiac hypertrophy in mice. The homology modeling strategy has shown that the C-09 molecule can bind neuraminidase 1 with subsequent activity reduction of most cardiac hypertrophic genes, improvement in left ventricular mass, left ventricle posterior wall depth (LVPWd), and cardiac fibrosis. The study confirmed a protective effect of the antiviral neuraminidase inhibitors zanamivir and oseltamivir on both structural and hemodynamic heart parameters and on the development of cardiac fibrosis [19].

**Figure 2:**
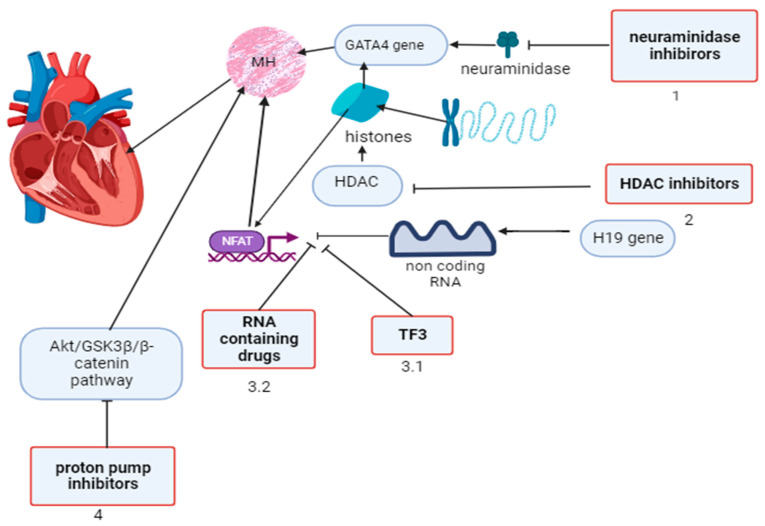
Modulation of gene expression pathways in myocardial hypertrophy and possible targets for available drugs. 1. Neurominidase inhibitors prevent the development of cardiac hypertrophy by preventing the interaction of enzymes with a nuclear GATA4 gene. 2. HDAC inhibitors prevent the expression of genes for the transcription factors GATA4 or NFAT by binding to them. 3.1. TF3 reducing calcineurin levels leading to inhibition of calcineurin–NFAT pathway activation. 3.2. RNA preparations, together with the H19 gene encoding the lncRNA, are modulators of NFAT expression and prevent myocardial hypertrophy. 4. Proton pump inhibitors suppress the upregulated Akt/GSK3β/β–catenin pathway, inhibiting myocardial remodeling. MH—myocardial hypertrophy; NFAT—nuclear factor of activated T-cell pathway; HDAC—histone deacetylase; TF3—theaflavin-3,3′-digallate. This image was generated using Biorender (accessed on 24 March 2024).

Lansoprazole, a proton pump H^+^/K^+^ adenosine triphosphatase (ATPase) inhibitor, was shown to suppress pathological cardiac remodeling in mice after transverse aortic constriction by inhibiting the upregulated Akt/GSK3β/β–catenin signaling pathway without affecting blood pressure and angiotensin II receptors by inhibiting angiotensin-stimulated cardiomyocytes and fibroblast remodeling independently of the gastric proton pump and inhibiting oxidative stress by increasing hem oxygenase expression [20]. Therefore, the modulation of myocardial hypertrophy based on gene expression pathways for neuraminidase and HDAC inhibitors, RNA preparations, theaflavin, and proton pump inhibitors seems to be promising for translation into practice (Figure 2).

## 3. Neonatal Posthypoxic Myocardial Hypertrophy, Factors of Gene Expression Regulation and Pharmacological Modulation

Posthypoxic hypertrophic cardiomyopathy is detected in 62.2% of neonates with perinatal hypoxia, and echocardiography is recommended during the first week of life. Myocardial hypertrophy is detected on echocardiography in 51% of newborns, of whom 69% showed intraventricular septal enlargement. These changes are associated with increased troponin T levels. Moreover, it was shown that left ventricular mass was two times more in the first 3 months after birth in preterm compared to term counterparts. These changes were associated with low gestational age, and cardiovascular problems could be seen later in these children [21].

Fetal hypoxia results in impaired autonomic regulation of the coronary vasculature, impaired energy metabolism (lack of ATP, ADP, and creatine phosphate), dysregulation of the Krebs cycle, activation of anaerobic glycolysis and impaired mitochondrial ultrastructure, both in cardiomyocytes and in cells of the conducting system [22]. In the mouse model of perinatal hypoxia, transcriptomic and methylomic data showed the state of fetal hypermethylation compared to adult hypomethylation [23].

Perinatal hypoxia (PH) in the postnatal period was found to significantly alter gene expression in congenital cyanotic heart disease in an experimental rodent model. In the upregulated state, the detected genes are responsible for cell cycle regulation, oxidative phosphorylation (subunit of cytochrome oxidase), glycolysis and anaerobic glycolysis, contractile function, sodium transmembrane influx, transcriptional activation (apoptosis in abnormal cells with reduced adhesion), and general apoptosis. Downregulated genes were those involved in collagen fibril assembly, excitation–contraction coupling, mitochondrial function, potassium transmembrane influx, oxidative phosphorylation (mitochondrial membrane ATP synthase), SERCA pump (sarcoplasmic reticulum calcium ATPase), L-type Ca++ channel, endogenous inotropy, gap junction cell–cell interaction, transcriptional activation (fatty acid storage and glucose metabolism), contractile force, and sarcomere structure. These changes, with no differences in septal and ventricular wall thickness, were associated with reduced contractility and reduced ejection fraction [24].

Prenatal hypoxia alters gene expression, activating stress-adaptive pathways, i.e., endoplasmic reticulum stress pathway, leading to apoptosis and cardiomyocyte degeneration [25]. In a rat model of newborn hypoxia, cardiomyocyte proliferation demonstrated a significant decrease with a resultant significant reduction in cardiomyocyte number and increasing of hypoxia-inducible 1 alpha (HIF-1alpha) transcription factor and pre-proET-1 (endothelin-1) mRNA elevation. Anoxia does not affect the binucleation or size of cardiomyocytes. Administration of ETA-receptor antagonists increases the proliferation of cardiomyocytes and myocardial mass and significantly decreases the effects of hypoxia [26]. If inadequately treated, focal dystrophy leads to focal cardiosclerosis and may be the result of hypoxic heart damage. Our studies showed that in 2-month-old rats subjected to perinatal hypoxia (PH), there is preexcitation in cardiac rhythm and a significant dominance of parasympathetic innervation in the regulation of cardiac electrical activity. We cannot exclude that the decrease in HR after PH was caused by sinus blockade, which may also reflect parasympathetic regulation of the heart rather than normal sympathetic control of electrical activity.

Prolongation of ventricular electrical systole, which may be caused by impaired ventricular myocardial conduction, was observed in the development of abnormalities in cardiac bioelectrical activity after PH. Under these conditions, the ventricular electrical repolarizing force increased 5.5-fold, indicating significant problems in restoring the membrane potential of the ventricular cardiomyocytes. We obtained data on the efficacy of metabolitotropic cardioprotectors thiotriazolin (thiazotic acid), angiolin, mildronate, and arginine in correcting post-PH cardiac bioelectric dysfunction. In normalizing the electrical activity of the heart and restoring the neurogenic regulation of the sinus node automaticity function, angiolin (a combination of metabolitotropic cardioprotector thiotriozolin and L-lysin) was shown to be the most effective.

Current evidence suggests that endothelial dysfunction and associated abnormalities in the NO system underlie the development of many cardiovascular diseases. Under the influence of hypoxia, infection, and other harmful factors, nitroxidergic system functioning is disturbed, accompanied by the development of pathology of organs and systems, including the cardiovascular system. The role of the NO system in the development of neonatal cardiovascular pathologies and the potential cardioprotective effects of modulating the NO system is poorly documented in the literature. Suppression of endothelial nitric oxide synthase (eNOS) expression, increase in the expression of its inducible form, and activation of nitrosative stress were found to be persistent disturbances of the cardiac nitroxidergic system after PH [27]. The abnormalities we found are consistent with modern views of myocardial damage mechanisms during ischemia and hypoxia. PH is known to reduce cardiac tolerance to ischemia/reperfusion, impair endothelial vasodilatation/vasoconstriction mechanisms, and contribute to cardiovascular pathology, including hypertension, atherosclerotic vascular disease, and heart failure [28].

There is evidence in the literature that suggests reduced eNOS expression and activity in cardiomyocytes and endothelium, as well as a risk of endothelial dysfunction following intrauterine hypoxia. Disturbances in eNOS activity could be explained by changes in interactions between eNOS and its regulatory partner proteins, such as caveolin-1, calmodulin, and HSP90. Alterations in phosphorylation and dephosphorylation of key eNOS serine and threonine residues may also contribute to eNOS dysfunction [29,30]. Endothelium-dependent vasodilation was impaired in coronary arteries of male and female offsprings subjected to PH at 4 and 9.5 months of age. This was associated with reduced eNOS and impaired SKCa and IKC channel function [31].

Low levels of eNOS are associated with impaired NO-dependent regulation of glutathione synthesis and reduced resistance to oxidative stress [32]. Decreased eNOS may be linked to hypoxia-inducible factor 1 alpha subunit (HIF-1a) deficiency. This factor activates inducible nitric oxide synthase (iNOS) expression by phosphorylating a serine residue [33]. Increased expression of iNOS was found to compensate for the decrease in eNOS after PH [34]. However, this leads to the formation of cytotoxic NO derivatives in “parasitic” reactions under conditions of reduced thiol antioxidant deficiency. Such reactions may occur under conditions of L-arginine deficiency, antioxidant deficiency, mitochondrial dysfunction, and increased iNOS expression. Uncontrolled formation of cytotoxic NO derivatives leads to nitrosylation of the most active sites of protein structures of ion channels, receptors, transmembrane pores, and signaling molecules, i.e., the development of nitrosative stress. An equally important consequence of myocardial ischemia is the loss of NO-mediated effects, such as suppressing cell proliferation, platelet aggregation, and, most importantly, inhibiting monocyte activation by so-called adhesion molecules [35].

Nitrosative stress also leads to heat shock protein 70 (HSP70) deficiency in the cell by depriving the glutathione linked to the thiol-disulphide system. Cytotoxic forms of NO not only lead to the modification (reversible and irreversible) of macromolecules, including HSP70 itself, but also reduce the expression of the genes encoding its synthesis [36,37]. Through suppression or induction, NO derivatives have been shown to regulate signaling pathways and associated gene activity [38]. Excess forms of nitric oxides, such as peroxynitrite and nitrosonium ion, first nitrolyze the thiol-redox-dependent regions of these genes and then oxidize them at increasing concentrations. Furthermore, cytotoxic forms of NO have a direct toxic effect on the myocardium, activating the processes of interstitial growth and fibrosis, increasing the negative inotropic effect of NO on the myocardium. Peroxynitrite can inhibit the mitochondrial electron transport chain by oxidizing thiols and binding to iron in cytochromes, exacerbating myocardial energy metabolism disorders [39].

NO in target cells can form active derivatives, such as nitrosonium (NO+), nitroxyl (NO-), and peroxynitrite (ONOO-). Recent studies have shown that NO and, in particular, its transformation products, such as peroxynitrite (ONOO-), nitrosonium ion (NO+), nitroxyl (NO-), and diazotrioxide (N2O3), are the main factors in the implementation of nitrosative stress, resulting in direct interaction of NO with metals (hemoglobin, hem iron, myoglobin, iron-containing enzymes, and non-hem iron of iron–sulfur proteins, and DNA), and indirect interaction of NO with myoglobin, iron-containing enzymes, copper, and zinc of enzyme active centers and NO+ (S-, N-, O-nitrosation) with thiol, phenol, hydroxyl, and amino groups of proteins and DNA [40]. Therefore, the use of the NO substrate of L-arginine has been the focus of researchers and clinicians for the reduction of the adverse effects of PH [41]. Our studies have shown a positive effect of arginine on the parameters of the NO system in the heart of 1- and 2-month-old rats after PH.

Pharmacological agents that combine the properties of positive modulators of NO and its transporters are promising. Thiotriazoline prevents the irreversible inactivation of the transcription nuclear factor kappaB (NF-kB) by protecting the sensitive cysteine residues Cys 252, Cys 154, and Cys 61 in its DNA binding domains from the excess of ROS. Thiotriazoline may participate in reducing these groups during reversible inactivation by acting as Redox Factor-1. Thiotriazoline enhances the activation of the expression of redox-sensitive genes, which are essential for cellular defense against oxidative stress. Thiotriazoline increases eNOS activity and reduces the intensity of nitrosative stress [42]. Thiotriazoline can increase NO bioavailability over-reactive oxygen species (ROS), is an antioxidant and ROS scavenger, and NO increases the activity of glutathione-dependent enzymes as well as the level of reduced glutathione during myocardial ischemia. ROS also stimulates cellular apoptosis-signaling kinase-1. This is a redox-sensitive kinase upstream of c-Jun N-terminal nuclear kinase (JNK) and p38 (mitogen-activated protein kinases family). Overexpression of apoptosis signal kinase-1 activates nuclear factor NF-kB to stimulate hypertrophy, whereas genetic suppression of apoptosis signal kinase-1 inhibits hypertrophy induced by angiotensin II, norepinephrine, and endothelin 1 [43,44,45]. All this suggests and justifies the use of pharmacological agents—modulators of the NO system for the protection of the myocardium after PH. 

**Figure 3:**
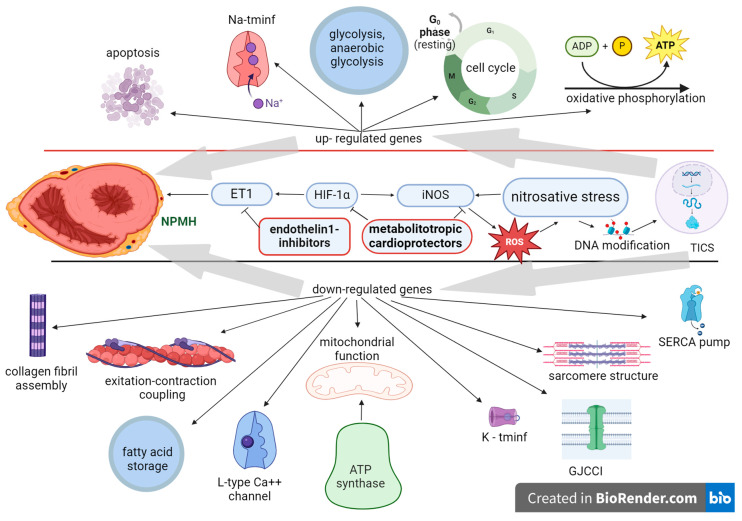
Endothelin-1 (ET1) inhibitors and metabolotropic cardioprotectors (MCs) are involved in the gene activity modulation of neonatal posthypoxic myocardial hypertrophy (NPMH) development. In NPMH, nitrosative stress affects DNA modification (DNAm) and transcription-induced cell signaling (TICS), altering gene expression. MCs inhibit HIF-1alpha-induced ET-1 vasoconstriction and iNOS, preventing DNAm, endothelin-1 inhibitors—auxiliary inhibit ET-1. Na-tminf—sodium transmembrane influx channel; K-tminf—potassium transmembrane influx channel; GJCC—gap junction cell–cell interaction; ROS—reactive oxygen species. This image was generated using BioRender (accessed on 24 March 2024).

The antioxidant mechanism of action of thiotriazolin consists of neutralizing ROS and cytotoxic products of NO, regulating ROS-dependent transcriptional processes by acting on NF-kB. Therefore, the metabolitotropic cardioprotectors with endothelin-1 (ET1) inhibitors, by influencing both the ET1-induced vasoconstriction and the nitrosative stress, reduce the main effects of PH on the myocardium (Figure 3). As shown in Figure 3, downregulated gene expression was mainly related to the integrity of the myocardium, whereas the upregulated genes were related to energy metabolism.

## 4. Hypertrophic Cardiomyopathy, Transcriptional Signaling Pathways, and Options for the Treatment

Current pharmacological or interventional treatments for patients with hypertrophic cardiomyopathy (HCM), although often effective in alleviating or preventing symptoms, do not target the underlying genetic defect or the key intermediate pathways underlying the phenotype. Therefore, this treatment is ineffective in the prevention or regression of cardiac hypertrophy and fibrosis. The development and testing of many pharmacological interventions are driven by the understanding of the molecular genetics and pathogenesis of HCM. Preliminary studies in animal models of hypertrophic cardiomyopathy have shown potential benefits of angiotensin II receptor blockers, statins, mineralocorticoid receptor blockers, and N-acetylcysteine.

There are 9 chromosomal loci associated with the condition, i.e., beta-myosin heavy chain, essential and regulatory myosin light chains, troponin T and I subunits, alpha troponin, cardiac myosin-binding protein C, cardiac actin, and titin. Mutations in sarcomeric genes (e.g., troponin1; MyBP-C (making cardiac myosin-binding protein C); TNNT2 (gene provides instruction for making troponin T); TTN (provides instructions for making very large protein titin); and myospryn (calcineurin interacting protein)) were described in primary non-syndromic hypertrophic cardiomyopathy (HCM). These genes encode proteins that are involved in the mechanism or control of contraction; therefore, hypertrophic cardiomyopathy is classified as a cardiac sarcomere disease [46,47]. More than 107 mutations have been identified in sarcomeric genes, and more than half of them are found in the beta-myosin heavy chain (MyBP-C3) gene. Some mutations are associated with a favorable prognosis, while others are associated with a high incidence of sudden cardiac death and severe hypertrophy [48].

Heterozygous frameshift mutations in MyBP-C3 result in hypertrophic cardiomyopathy. Long-term gene therapy using MyBP-C3 in homozygous mice is underway. Delivery of MyBP-C3 using an adeno-associated virus resulted in the prevention of hypertrophic cardiomyopathy in mice. MyBP-C3 gene therapy unexpectedly also suppresses the accumulation of mutant mRNAs. The first successful long-term gene therapy for HCM correcting both haploid insufficiency and noxious peptide production is reported in this study [49]. Interfering RNAs targeting the mutant allele of MyBP-C3 are also under investigation. For example, administration of iRNA delays the manifestation of cardiac hypertrophy and fibrosis [50].

Another cause of HCM is a group of conditions called RASopathies, or non-sarcomeric HCM, caused by mutations in the RAS/MAPK signaling pathway, which controls cell proliferation, differentiation, migration, and apoptosis. RASopathies are present in Noonan syndrome, Noonan syndrome with multiple lentigines (NSML), Costello syndrome, cardiofaciocutaneous syndrome, neurofibromatosis type 1, and Legius syndrome [51]. RAF1 mutations (regulation of the RAS/MAPK signaling pathway) are associated with Noonan and NSML as the cause of HCM in 65% of patients [52]. RAS/MAPK activation leads to myocardial hypertrophy and cardiac fibers disorganization [53]. Treatment with MEK1 (mitogen-activated protein kinase belonging to the RAS/MAPK) inhibitors (kinase inhibitors) reduces the development of HCM caused by RIT1 (RAS/MAPK activator) mutation in rats [54]. A missense mutation of the PTPN11 (protein tyrosine phosphatase nonreceptor type 11) gene is another RAS/MAPK regulator that produces the SHP-2 (small heterodimer partner, intracellular transcription factor) protein and is associated with NSML and HCM. This mutated gene increases the activity of the mTOR (mammalian target of rapamycin)-PI3K (phosphoinositide 3 kinases)-AKT (makes AKT1 kinase protein, regulation of cell growth, proliferation, and differentiation) signaling pathway, which is important in regulating the cell cycle. Signs of HCM are reduced by mTOR-PI3K-AKT inhibitors, such as rapamycin (an immunosuppressive drug) [55].

Recently, an infant patient with rapidly progressive HCM in NSML and evidence of PTPN11 mutation was treated with rapamycin analog, and cardiac improvement was demonstrated after 12 weeks of low-dose administration [56]. N-acetylcysteine (NAC), a drug from the group of thiol antioxidants with properties of ROS and nitric oxide scavenging, increases glutathione levels and activity of glutathione-dependent enzymes (glutathione peroxidase and glutathioreductase), reduces iNOS overexpression, and regulates IL-1b receptor sensitivity. NAC reduced cardiac fibrosis and associated ventricular hypertrophy in male neonates on the background of maternal obesity, possibly by reducing oxidative stress. Moreover, NAC restored normal Akt-mTOR signaling in offspring [57]. Administration of NAC to β-MyHC-Q403 transgenic rabbits and cTnT-Q92 transgenic mice normalized myocardial and blood levels of oxidized glutathione. It reversed cardiac hypertrophy and interstitial fibrosis and prevented left ventricular systolic dysfunction [58].

Mitochondrial dysfunction plays an important role in the formation of hypertrophic cardiomyopathy. Several proteins are responsible for the integrity of mitochondrial structure and function. Most of them are encoded by nuclear DNA (nDNA), and only a small proportion by mitochondrial DNA (mtDNA) [59]. As the heart muscle is one of the tissues that requires much energy, damage to the heart muscle (i.e., mitochondrial cardiomyopathy) occurs in about 20–40% of children with mitochondrial diseases. Heart damage can occur as an isolated feature or as a part of a multi-organ lesion [60]. In addition to the classic mitochondrial syndrome, several other mitochondrial disorders have been identified in association with HCM, which can be classified as mitochondrial diseases caused by deficiency of one or more respiratory chain complexes, i.e., CoQ10 (coenzyme Q10) deficiency, mitochondrial transporter deficiency, the disorders characterized by 3-methylglutaconium aciduria (e.g., Bart’s syndrome), and disorders of mitochondrial iron metabolism (e.g., Friedreich’s ataxia) [61]. In mitochondrial cardomyopathies (MCMs), the involvement of different molecular pathways takes place, resulting in the formation of hypertrophic, dilated, and arrhythmogenic cardiomyopathies development.

Mice deficient in the *Amt1* gene (adenine nucleotide translocator type 1) show myocardial hypertrophy and proliferation of mitochondria, similar to the MCM seen in humans. Mutations in *Med30* (mediator of RNA polymerase II transcription subunit 30) negatively affect the transcription of genes involved in oxidative phosphorylation, resulting in progressive cardiomyopathy. Inhibiting mitochondrial transcription factor A (TFAM) damages oxidative phosphorylation and increases ROS, leading to cell cycle inhibition. Mutations in CHCHD10 (coiled-coil helix domain/chain, activate transcription in hypoxia and normoxia) result in oxidative stress, iron dysregulation, and mitochondrial dysfunction. In the management of MCM along with standard symptomatic therapy, the pharmacological approach considered is resveratrol and epicatechin. These polyphenols are potent antioxidants that activate sirtuin 1–3 genes (deacetylate mitochondrial proteins regulate ATP production) and eNOS, PGC1 alpha (peroxisome proliferator-activated receptor-gamma coactivator and mitochondrial transcription factor), and NRFs (nuclear respiratory factors). Nicotinamide riboside, given as a dietary supplement, was found to activate sirtuin 1 and reduce cardiac hypertrophy in a mouse model [62].

**Figure 4:**
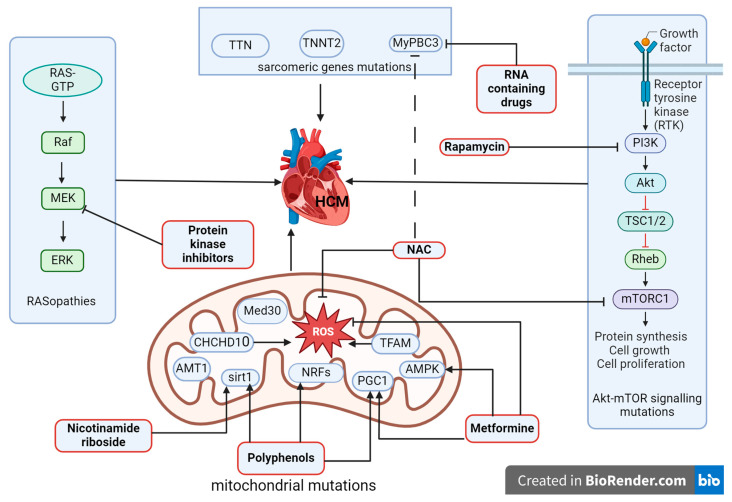
Signaling pathways. Sarcomeric and mitochondrial genes involved in the development of HCM and their pharmacological modulation. N-acetylcysteine (NAC) restores Akt-mTOR signaling and, together with RNA-containing drugs, inhibits MyBP-C3 and oxidative stress. Metformin potentiates AMPK and PGC1 with polyphenols and inhibits oxidative stress; polyphenols also potentiate NRFs and activate SIRT1 with nicotinamide riboside, and protein kinase inhibitors act on MEK1 (mitogen-activated protein kinase) in the RAS/MAPK pathway. HCM—hypertrophic cardiomyopathy; MyBP—C3-beta-myosin heavy chain; TNNT2—troponin T; TTN—titin; NAC—N-acetylcysteine; ROS–reactive oxygen species; TFAM—mitochondrial transcription factor A; Med30—mediator of RNA polymerase II transcription subunit 30; AMPK—adenosine monophosphate-activated protein kinase; AMT1—adenine nucleotide translocator type 1; SIRT1—sirtuin 1; NRFs—nuclear respiratory factors; CHCHD10—coiled-coil helix domain/chain 2; PGC1-1 alpha—peroxisome proliferator-activated receptor-gamma coactivator. This image was generated using BioRender (accessed on 24 March 2024).

Metformin has been shown to enhance AKT (A-kinase C-terminal domain, protein kinase B) and activate RISK (reperfusion injury salvage kinases, responsible for cardioprotection in reperfusion) pathways, restore decreased PGC1 alpha levels, increase eNOSser1177 phosphorylation, and improve mitochondrial metabolism as well as cardiovascular injury after ischemia. Metformin supports myocardial energy metabolism by activating the AMPK pathway (adenosine monophosphate-activated protein kinase) and controlling lipid and glucose metabolism in cardiomyocytes, increasing NO bioavailability, limiting interstitial fibrosis and cardiomyocyte apoptosis in ischemia, and improving the autophagic function of the aging heart muscle [63,64,65]. Thus, pharmacological agents could have a differential impact on specific pathogenetic mechanisms involved in the development of HCM (Figure 4).

## 5. Conclusions, Discussion, and Future Perspectives

The experimental data analyzed by us, as well as some clinical data from recent studies, reflect the possibilities available for the application of known pharmacological agents that modulate different signaling pathways in some conditions associated with hypertrophic processes in the heart. It should also be noted that these agents, although reasonably available, are not part of standard treatment programs. At the same time, there are insufficient data on the clinical efficacy of these modulators of gene transcriptional pathways activated in myocardial hypertrophy, especially in children. In our review, we outlined both directions on further clinical studies of practical applicability and the options for combining different remedies, taking into account the points of their application regarding the specific signaling pathways involved in the pathological process.

In our opinion, this approach is most relevant to slowly developing hypertrophic processes in the myocardium with little or no manifestation, which is most common in childhood. For example, in children with BAV with slowly progressive left ventricular hypertrophy and calcineurin pathway (NFAT) gene activation, noncoding RNAs show significant inhibitory activity. Using RNAi-based drugs is considered one of the most promising areas, including heart diseases [66]. The same signaling pathway is inhibited by theaflavin, which is naturally derived and could be safely used in addition to RNA drugs in the future. Neuraminidase inhibitors, widely used as antivirals, also inhibit GATA signaling in the heart and could, therefore, be considered for viral heart damage (e.g., myocarditis), which is quite common in childhood. HDAC inhibitors are synergistic with neuraminidase inhibitors for the GATA pathway and with RNAi drugs and theaflavin for the NFATC pathway. However, the most specific of these, such as hydroxamic acid, benzamides, and cyclic peptides, may not be promising because of their safety profile and side effects, although short-chain fatty acids, being weak inhibitors, may be applicable in children with myocardial hypertrophy in combination with inhibitors of other specific pathways.

Proton pump inhibitors (PPIs) are of particular interest in this context. In addition to their well-known role as regulators of gastric secretion, PPIs can directly influence myocardiocyte proliferation and remodeling by inhibiting the Akt/GSK3β/β–catenin signaling pathway independently of gastric proton pump. Despite the predominantly experimental data, PPIs can be regarded as a promising approach, especially given the comorbid hyperacidity in pediatric patients.

In NPMH, the experimental data indicate that many genes and their activated pathways are involved in the pathological process and that their expression is altered by oxidative stress. The downregulated genes were mostly related to myocardial structure and integrity, while the upregulated genes were associated with compensatory activation of energy metabolism, cell cycle, and apoptosis. Depending on the intensity and duration of hypoxic exposure, these changes are generally considered dystrophic. In this respect, there may be a large number of metabolitotropic cardioprotective agents available, including in the neonatal period.

Given the safety profile of these agents, the derivatives of thiazotic acid in combination with L-arginine and L-lysine may be considered optimal for use in this category of patients. In addition, although endothelin-1 inhibitors require further study of their clinical efficacy in children, they can be considered a promising direction of therapy in combination with metabolitotropic cardioprotectors, potentiating antihypoxic effects and enhancing cardiomyocyte proliferation. On this basis, these agents may have a regulatory and normalizing effect on gene expression under hypoxic conditions by inhibiting nitrosative stress-induced DNA modification.

In HCM, the clinical evidence for the efficacy of long-term low-dose administration of immunosuppressive drugs (e.g., rapamycin), although used in one pediatric patient, reflects areas for future study regarding reduced immunosuppression and specific inhibition of the Akt-mTOR signaling pathway in the heart. In sarcomeric HCM, the RNA-containing drugs may specifically inhibit the mutated MyBP-C3 gene, preventing myocardial hypertrophy and fibrosis. In RASopathies (syndromic HCM), protein kinase inhibitors reduce the MEK activity of RAS/MAPK signaling in the heart. However, these agents belong to the antitumor class with systemic cytostatic effects and have fewer prospects for use in pediatric HCM. In mitochondrial HCM, mutations in mitochondrial genes with systemic phenotypic involvement and cardiac damage lead to impaired energy metabolism and cause oxidative stress that increases ROS. Metformin has been found to have multiple effects on mitochondrial function, affecting key signaling pathways of AMPK and PGC1, as well as reducing ROS.

Polyphenols have a synergistic effect on PGC1 and, with nicotinamide riboside, also on SIRT1. Overall, these agents potentiate normal mitochondrial signaling and may compensate for altered gene function in mitochodriopathies. NAC has a threefold role in this context, affecting Akt-mTOR signaling, MyBP-C3 function in sarcomeric HCM, and reducing oxidative stress. Because of its safety and minimal side effects, NAC can be used in combination with RNA drugs and metformin in various forms of HCM. Non-selective drugs (beta-blockers, calcium channel blockers, antiarrhythmics, ACE II receptor antagonists, valsartan, and trimetazidine) have been proposed for the treatment of HCM.

Specific drugs known as myosin inhibitors (mavacamten and aficamten) have passed the first stage of clinical trials in adults and are targeting the structure of the sarcomere in acquired HCM [67]. In this review, we have summarized recent breakthroughs in modulating gene signaling pathways, including mutations involved in some types of genetic heart conditions development, focusing on available and relatively safe pharmacological agents that allow them to be combined for specific hypertrophic myocardial changes in children and to translate these data in future clinical studies. A summary of the data can be found in Table 1.

Table 1 summarizes the potential for the selective and combined use of safe and affordable classes of drugs against specific gene signaling pathways in different myocardial hypertrophic processes, potentially expanding therapeutic options in pediatric practice.

## Figures and Tables

**Table 1:** The most common hypertrophic cardiac diseases in children, associated gene signaling pathways, and potential modulating pharmacological agents.

Myocardial Changes in Children	Signaling Pathways	Modulating Pharmacological Agents
Myocardial hypertrophy in congenital heart diseases	GATA4	Neuraminidase inhibitors
	HDAC	HDAC inhibitors
	NFAT	RNA-containing drugs, theaflavin-3,3′-digallate
	Akt/GSK3β/β–catenin	Proton pump inhibitors
Neonatal posthypoxic hypertrophic myocardial cardiomyopathy	ET1	Endothelin 1 inhibitors
	HIF1α	Metabolitotropic cardioprotectors
Genetic hypertrophic cardiomyopathy	MyBP-C3	RNA-containing drugs, N-acetylcysteine
	Akt-mTOR	Rapamycin, N-acetylcysteine
	AMPK, PGC1	Metformin, polyphenols
	NRFs, SIRT1	Polyphenols, Nicotinamide riboside
	RAS/MAPK	Protein kinase inhibitors
